# Global pharmacovigilance regulations: Call for re-harmonization

**DOI:** 10.1177/1740774518801592

**Published:** 2018-09-29

**Authors:** Ajay Singh, Ken Twomey, Robert Baker

**Affiliations:** 1 GlaxoSmithKline, Collegeville, PA, USA; 2 AstraZeneca UK Limited, Cambridge, UK; 3 Lilly Corporate Center, Eli Lilly and Company, Indianapolis, IN, USA

April 1990 marked the inception of the International Council for Harmonisation (ICH), an organization created through the joint efforts of regulatory authorities and pharmaceutical companies, with the mission to align global pharmaceutical regulatory requirements. Over the ensuing quarter century, among other accomplishments, harmonized processes for submission of new drug applications and standardized coding of adverse events have been accepted globally.^1^ Such harmonization, which has facilitated the collaboration of research on a global basis, is particularly critical when characterizing and communicating the risk profile of drugs (field of pharmacovigilance). Of late, however, TransCelerate, a collaborative effort of 19 member companies,^2^ including most of the largest pharmaceutical companies, has identified a concerning trend of diverging regulations with regard to handling of pharmacovigilance findings from ongoing clinical trials.

Examples include the processes for determining whether adverse events reported by investigators are related to investigational drug and expected (i.e. consistent with the known safety profile of the product and/or events anticipated in the population). Unexpected and related serious adverse events may be subject to expedited reporting to apprise investigators and regulators of potential risks. Recent European guidelines require comprehensive expedited reporting of serious events,^3^ while US Food and Drug Administration (FDA) guidance restrict reporting to key (sponsor-adjudicated) related events.^4^ While in isolation, the rationale for each of the above guidelines is meritorious, the divergence of these requirements is complicating consistent communication of safety profiles to stakeholders.

Over the last two decades, the clinical trial landscape has evolved considerably: (1) many more countries are participating; (2) diverse populations, often with significant underlying comorbidities, are being evaluated; and (3) new technologies are being developed. Consequently, optimal benefit–risk assessment requires close collaboration between all stakeholders from the investigators with their deep expertise of the disease and individual patient data, to the sponsor with their in-depth knowledge of the specific molecules and to the various regulatory authorities who oversee safety and efficacy of drugs from various sponsors. To that end, communication of congruous safety data (by sponsors) to other stakeholders has become especially important; in this way, investigators and regulators across all regions will have comparable insight into the evolving safety profile of new products. To meet these goals, we at TransCelerate implore representatives for regulatory authorities to work with industry sponsors, in the spirit of ICH, to identify potential ways to re-harmonize global pharmacovigilance processes and requirements.
